# Avian Influenza H5N1 in Naturally Infected Domestic Cat

**DOI:** 10.3201/eid1204.051396

**Published:** 2006-04

**Authors:** Thaweesak Songserm, Alongkorn Amonsin, Rungroj Jam-on, Namdee Sae-Heng, Noppadol Meemak, Nuananong Pariyothorn, Sunchai Payungporn, Apiradee Theamboonlers, Yong Poovorawan

**Affiliations:** * Kasetsart University, Nakorn Pathom, Thailand;; † Chulalongkorn University, Pathumwan, Thailand;; ‡ Western Veterinary Research and Development Center, Chombueng, Ratchaburi, Thailand

**Keywords:** Avian Influenza, H5N1, cat, dispatch

## Abstract

We report H5N1 virus infection in a domestic cat infected by eating a pigeon carcass. The virus isolated from the pigeon and the cat showed the same cluster as the viruses obtained during the outbreak in Thailand. Since cats are common house pets, concern regarding disease transmission to humans exists.

Highly pathogenic avian influenza (HPAI) H5N1 causes death in many avian species and mammals, including humans (*1**–**5*). In Thailand, infection by HPAI H5N1 has been reported in mammalian species such as tigers (*1**,**3*) and cats (*6*). Most infected mammals had high fever, panted, and showed symptoms of depression, myalgia, and nervousness (*4*). This article reports H5N1 infection in a cat during the early H5N1 outbreaks in Thailand and characterizes the genome of H5N1 virus isolated from the infected domestic cat.

## The Study

**Figure 1:**
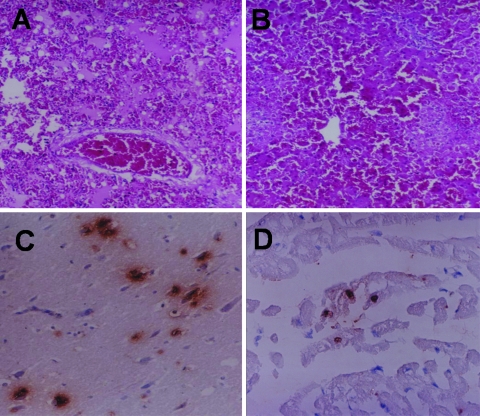
Microscopic lesions of the infected cat, lung edema with homogeneous pink material and congestion (A) and multifocal necrosis in the liver (B). Positive sites are shown by immunohistochemical examination of the infected cat in neurons (C) and cardiac muscle cells (D) (magnification ×100).

In early February 2004, during the outbreak of HPAI (H5N1) in Thailand, a carcass of a 2-year-old male cat (*Felis catus*) was taken in an icebox 6 hours postmortem to the Faculty of Veterinary Medicine at Kasetsart University, Nakornpathom, Thailand. The cat's owner volunteered the information that the cat had eaten a pigeon (*Columba levia*) carcass 5 days before illness onset. The owner reported that the cat had a temperature of 41°C, was panting, and appeared to be depressed. Furthermore, the cat had convulsions and ataxia and died 2 days after onset of illness. The cat was given a single dose of 75 mg aspirin 1 day before it died; however, its body temperature remained elevated. Many dead pigeons were found in the area where the cat lived. Necropsy of the cat showed cerebral congestion, conjunctivitis, pulmonary edema, severe pneumonia, renal congestion, and hemorrhage in the intestinal serosa. Tissues from brain, trachea, lungs, mesenteric lymph nodes, intestines (duodenum, jejunum, and ileum), kidneys, liver, pancreas, spleen, and heart were collected, fixed with 10% buffered formalin, and processed for histopathologic examination. Histopathologic examination results showed nonsuppurative encephalitis, gliosis, mononuclear infiltration into the Virchow-Robin space, vasculitis, and congestion in both cerebrum and cerebellum. A microscopic lesion in the lung was caused by severe pulmonary edema, interstitial pneumonia, and congestion (Figure 1A). Multifocal necrosis in the liver (Figure 1B), tubulonephritis, and lymphoid depletion in the spleen were found. No abnormalities were detected in any other organs.

The paraffin-embedded tissues, including brain, lung, kidney, heart, spleen, pancreas, liver, and intestine tissue, were examined immunohistochemically.A polyclonal goat anti-HPAI H5N1 (Kasetsart University, Nakornpathom, Thailand) diluted 1:400 in phosphate-buffered saline was used as the primary antibody. The secondary antibody was polyclonal mouse anti-goat immunoglobulin G (Zymed Laboratories, Inc., San Francisco, CA, USA) diluted 1:200 in phosphate-buffered saline. Diamino benzidine was the substrate developed as a chromogen. Tissue from a cat that had been hit and killed by a car was used as the negative control. Sites displaying a positive H5N1 antigen reaction were in cerebral neurons (Figure 1C), heart (myocardial cells) (Figure 1D), pneumocytes, renal tubular epithelial cells, hepatic cells, and white pulp of the spleen (macrophages). The pancreas and intestine were negative for H5N1 antigen.

Parts of frozen brain, lung, liver, kidney, spleen, and duodenum content were ground separately, and virus isolation testing was conducted by using embryonated egg injection. Virus isolation testing was also conducted on pleural fluid and urine. Virus isolation testing was conducted by injecting pleural fluid, urine, and filtrates obtained from the ground tissues into the allantoic sac of 10-day-old embryonated chicken eggs. Embryonic death occurred 18 hours after injection. The allantoic fluids of the dead embryos were subjected to hemagglutination (HA) and hemagglutination inhibition tests. All fluids from the dead embryos were positive for avian influenza A (H5). The virus could be isolated from all injected specimens. To identify the subtype, reverse transcription–polymerase chain reaction was conducted, and the virus was confirmed to be influenza A H5N1 (*7**,**8*). The HPAI H5N1 isolate recovered from the infected cat's lung was labeled A/Cat/Thailand/KU-02/04. In addition, an isolate of HPAI H5N1 from an infected pigeon in the area where the cat lived was included in the study and labeled A/Pigeon/Thailand/KU-03/04.

**Figure 2:**
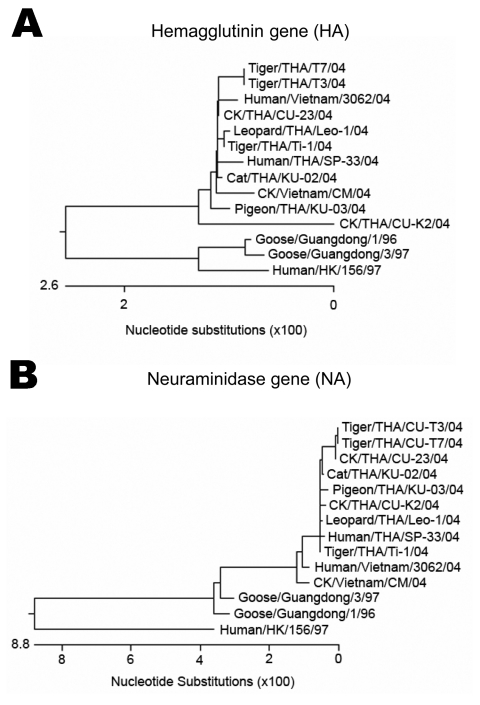
Phylogenetic analysis of the hemagglutinin (A) and neuraminidase (B) gene sequences of highly pathogenic avian influenza H5N1 from the cat in this study, compared with other sequences from GenBank database.

H5N1 viruses isolated from the cat's (KU-02) and the pigeon's (KU-03) lung tissue were characterized in this study. The entire genome sequence was determined in the H5N1 isolate from the cat, while the H5N1 isolate from the pigeon was sequenced to specifically obtain the HA, neuraminidase, and PB2 genes. The sequences obtained from the cat (H5N1) (A/Cat/Thailand/KU-02/04) were submitted to the GenBank database under accession numbers PB2 (DQ236079), PB1 (DQ236080), PA (DQ236081), HA (DQ236077), NP (DQ236082), NA (DQ236078), M (DQ236084), and NS (DQ236083). The sequences obtained from the pigeon (H5N1) (A/Pigeon/Thailand/KU-03/04) were submitted to GenBank under accession numbers HA (DQ236085), NA (DQ236086), and PB2 (DQ236087). Sequencing and phylogenetic analysis of the HA (Figure 2A) and NA (Figure 2B) genes of HPAI isolates (cat and pigeon) showed that the HA and NA genes of the viruses were similar to each other as well as to those of the viruses isolated from tigers, chickens, and humans in Thailand. Genetic comparisons of each gene of the cat isolate (KU-02) to those of the viruses isolated from chickens (January and July 2004) and tigers (January and October 2004) are shown in the Table. The analyses showed that the cat isolate (KU-02) was closely related to other H5N1 isolates collected from the region in 2004. This finding indicated that the H5N1 infection in the cat resulted from the virus circulating during the H5N1 outbreaks in early 2004. The HA gene of KU-02 and KU-03 contained multiple basic amino acid insertions at the HA cleavage site (SPQRERRRKKRR) as well as glutamine and glycine (Q222–G224) at the receptor binding site. The NA genes of KU-02 and KU-03 also had 20 amino acid deletions at positions 49–68 and contained histidine at position 274, indicating absence of antiviral drug resistant residues. The NS gene of the KU-02 isolate contained a 5–amino acid deletion (79–83), and the M2 gene of the KU-02 isolate contained an amino acid (asparagine) at position 31, conveying amantadine resistance. In summary, the viruses from the cat and the pigeon were similar to the H5N1 viruses isolated in Thailand and Vietnam in 2004, which had then been identified as genotype Z (*9*). A single amino acid substitution at position 627 of the PB2 gene (glutamic acid to lysine) was observed in the cat isolate (KU-02), as had previously been shown in the tiger isolates (*1*). In contrast, the PB2-627 amino acid residue of the pigeon isolate (KU-03) remained unchanged (glutamic acid).

**Table:** Genetic comparison of the 8 gene segments of the cat isolate (KU-02) to those of H5N1 isolates from Thailand

Gene	Region of comparison	% nucleotide identity					
		KU-03 Pigeon (Jan 2004)	CU-T3 Tiger (Oct 2004)	CU-23 Chicken (Jul 2004)	Ti-1 Tiger (Jan 2004)	Leo-1 Leopard (Jan 2004)	CU-K2 Chicken (Jan 2004)
HA	46–1623	99.6	99.7	99.9	99.9	99.8	98.5
NA	25–1297	99.6	99.5	99.6	99.9	99.8	99.8
M	1–952	–	99.9	99.8	99.6	99.5	99.7
NS	36–824	–	99.5	99.9	99.9	99.7	99.5
NP	58–1474	–	99.7	99.7	99.8	99.9	99.9
PA	28–2132	–	99.6	99.8	99.6	99.6	99.5
PB1	62–2226	–	99.8	99.8	99.8	99.8	99.7
PB2	82–2220	99.5	99.6	99.7	99.6	99.7	99.6

## Conclusions

This study is the first to report entire H5N1 genome sequences in a naturally infected domestic cat in Thailand, although experimental infection by H5N1 in domestic cats has been reported (*10*). The case of H5N1 in a cat was reported during the early H5N1 outbreaks in Thailand in February 2004. The likely route of infection was eating an infected pigeon carcass. Our study confirmed H5N1 infection in pigeon carcasses from the same area. In our study, both H5N1 isolates from the cat and the pigeon displayed characteristics identical to H5N1 isolates from the epidemic in Thailand. Moreover, genetic comparison indicated that the virus isolated from the cat (KU-02) was more similar to the H5N1 viruses from early 2004 (Ti-1 and Leo-1) than those from late 2004 (CU-T3 and CU-23).

Our results demonstrated that domestic cats are also at risk for H5N1 infection. Clinical signs and pathologic test results of the cat in this study are similar to those of an experimental study by Kuiken et al. conducted in 2004. Cats are companion animals and may live in very close contact with humans. Although no direct transmission of H5N1 from cats to humans has been reported, it is possible; therefore, cats in H5N1-endemic areas should be scrutinized. In *Felidae*, such as tigers and cats, probable horizontal transmission of H5N1 within the same species has been found (*4*). However, the risk for transmission from poultry to humans is probably much higher because poultry outnumber cats and excrete higher titers of the H5N1 virus (*10*). Hence, monitoring domestic animals for infection during H5N1 outbreak is recommended.
